# Clinical applications of 3D printing in colorectal surgery: A systematic review

**DOI:** 10.1007/s00384-024-04695-8

**Published:** 2024-08-07

**Authors:** Alyssa C. Habermann, William R. Timmerman, Stephen M. Cohen, Brian W. Burkhardt, Michael F. Amendola

**Affiliations:** 1 Department of Surgery, Virginia Commonwealth University, Richmond, USA; 2 Department of Surgery, Central Virginia Veterans Affairs Healthcare System, Richmond, USA; 3 Office of Advanced Manufacturing Site Lead, Department of Physical Medicine and Rehabilitation, Central Virginia Veterans Affairs Healthcare System, Richmond, USA; 4 Division of Vascular Surgery, Central Virginia Veterans Affairs Healthcare System, Richmond, USA

**Keywords:** 3D printing, 3D model, Colorectal surgery, Presurgical planning, Anatomy model

## Abstract

**Background:**

The utilization of three-dimensional printing has grown rapidly within the field of surgery over recent years. Within the subspecialty of colorectal surgery, the technology has been used to create personalized anatomical models for preoperative planning, models for surgical training, and occasionally customized implantable devices and surgical instruments. We aim to provide a systematic review of the current literature discussing clinical applications of three-dimensional printing in colorectal surgery.

**Methods:**

Full-text studies published in English which described the application of 3D printing in pre-surgical planning, advanced surgical planning, and patient education within the field of colorectal surgery were included. Exclusion criteria were duplicate articles, review papers, studies exclusively dealing with surgical training and/or education, studies which used only virtual models, and studies which described colorectal cancer only as it pertained to other organs.

**Results:**

Eighteen studies were included in this review. There were two randomized controlled trials, one retrospective outcomes study, five case reports/series, one animal model, and nine technical notes/feasibility studies. There were three studies on advanced surgical planning/device manufacturing, six on pre-surgical planning, two on pelvic anatomy modeling, eight on various types of anatomy modeling, and one on patient education.

**Conclusions:**

While more studies with a higher level of evidence are needed, the findings of this review suggest many promising applications of three-dimensional printing within the field of colorectal surgery with the potential to improve patient outcomes and experiences.

## Introduction

Traditionally, surgeons have used two-dimensional (2D) imaging modalities such as computed tomography (CT) or magnetic resonance imaging (MRI) to evaluate patient anatomy for the purposes of diagnostics and surgical planning. With the advent of three-dimensional (3D) printing technology came the potential for its application in surgical practices by providing surgeons with improved visual and tactile interaction with complex anatomy [1]. This replicates surgeons’ experience in the operating room, in which spatial relationships and touch contribute immensely to the ability to confirm anatomy and determine which structures can safely be altered or removed. Additionally, processing complex information for surgical planning can take less time with the aid of a 3D printed structure compared to traditional 2D imaging [2]. Accordingly, the number of medical centers in the United States with 3D printing capabilities is growing rapidly [3]. Initially, 3D printing was primarily embraced by subspecialties such as maxillofacial surgery, orthopedics, and neurosurgery, likely due to the complex bony reconstructions and frequent use of prostheses inherent to these specialties [4]. In more recent years, the field of general surgery has begun to adopt the technology for the benefit of both direct patient care and education for medical learners and patients.

In colorectal surgery specifically, 3D printing has been used to create personalized anatomical models, largely for preoperative planning, as well as models for surgical training [5]. There has been little in the way of implantable medical device design and/or advanced surgical planning, which denotes the actual utilization of 3D printed materials in the operative setting. Given the rapid evolution of the field, previous reviews of 3D printing in colorectal surgery are dated, missing current and relevant additions to the literature [5–7]. There is a recent and thorough paper discussing the role of 3D printing in surgical education with respect to colorectal surgery, thus the topic of trainee education will not be addressed in this paper [8]. Instead, this review will focus on the current clinical applications of 3D printing in colorectal surgery.

The aim of this review is to summarize the current clinical applications of 3D printing in colorectal surgery in order to understand current uses, gaps in the literature, and potential for future directions.

## Methods and materials

The Preferred Reporting Items for Systematic Reviews and Meta-Analyses (PRISMA) guidelines were used to conduct this systematic review [9]. The completed PRISMA checklist can be found in Appendix A. No funding was obtained for this study.

### Literature search

The electronic databases searched to identify studies for this review were PubMed/MEDLINE, Google Scholar, and Cochrane. Keywords in the search strategy included “3D printing,” “3D model,” “colorectal,” “colorectal surgery,” “rectal surgery,” “colorectal cancer,” and “inflammatory bowel disease,” as well as abbreviations and synonyms. MeSH terms were employed. The final search was performed on 10 August 2023.

### Inclusion criteria

All full-text studies published in English which described the application of 3D printing in pre-surgical planning, advanced surgical planning (intra-operative guidance), and patient education as they pertained to the field of colorectal surgery were included.

### Exclusion criteria

Duplicate articles, review papers, studies which exclusively dealt with surgical training and/or education, studies which used only virtual and not printed models, and studies which described colorectal cancer only as it pertained to other organs (e.g., liver metastases of colorectal cancer) were excluded.

### Screening and data extraction

Title and abstract screening were performed by one reviewer (Habermann A). Three additional reviewers (Amendola M, Cohen S, and Timmerman W) were presented the search results and assisted in determining which studies met inclusion criteria. Information extracted from each study utilized in this review included title, author, date of publication, patient demographics, indication for 3D modeling, type of 3D model, methodology of segmentation, time and cost of 3D modeling, clinical outcomes, surgeons’ evaluation of clinical utility, and patient satisfaction.

### Data analysis/synthesis

The results were divided into the following categories based on study purpose: advanced surgical planning/device manufacturing, pre-surgical planning, anatomical model (subdivided into pelvic anatomy model, vascular anatomy model, fistula anatomy model, ileal pouch anatomy model), and patient education. Advanced surgical planning refers to 3D printed models that are incorporated into the actual operation and not just used as a visual aid. Papers in each category were compared with regards to study type, imaging modality used, type of model created, manufacturing details, and cost. Metanalysis was not performed given the small number of studies in each category and the inconsistency of data reporting between papers.

## Results

**Fig. 1:**
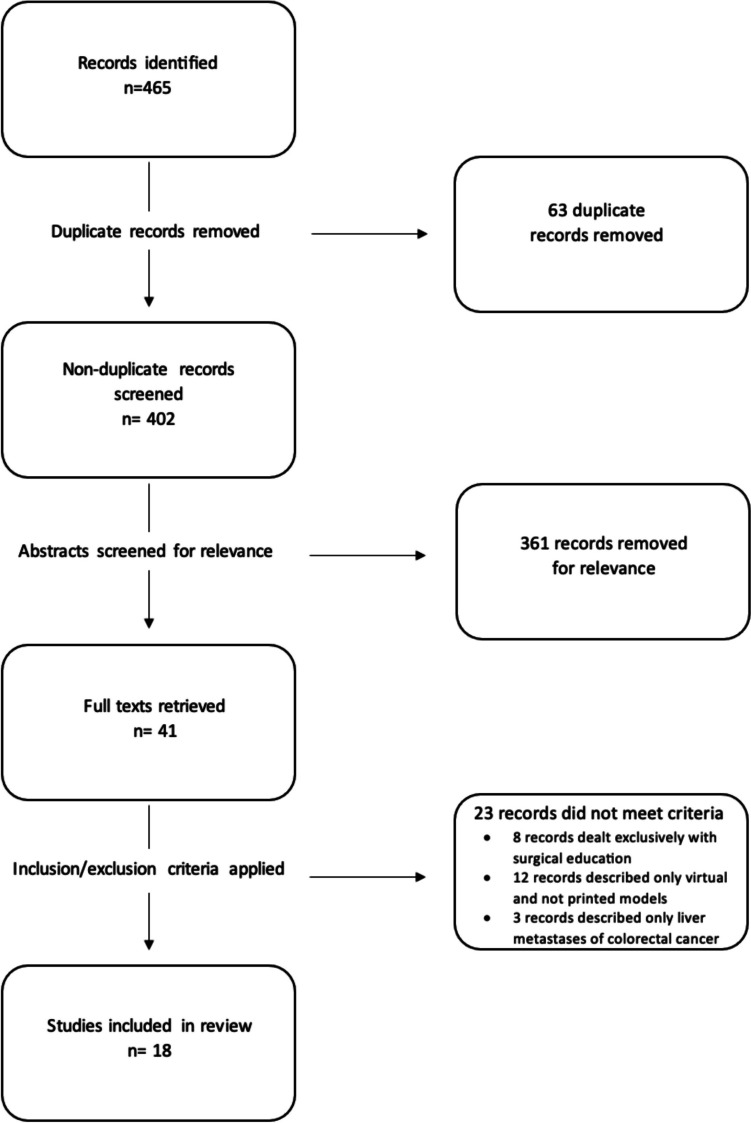
PRISMA flow chart

The search identified 465 papers, and after removing duplicates and screening for relevance, yielded 41 results. After applying the inclusion and exclusion criteria, 18 papers remained (Fig. 1). There were eleven studies from Asia and seven studies from Europe; there were not any studies from the United States or North America. The studies were published between 2016 and 2023. There were two randomized controlled trials, one retrospective outcomes study, five case reports/series, one animal model, and nine technical notes/feasibility studies. Models were derived from CT in twelve studies and MRI in three studies; in three papers models were not based on a 2D imaging modality. Further details regarding the details of 3D models and prints in these studies can be found in Table 1.

**Table 1:**
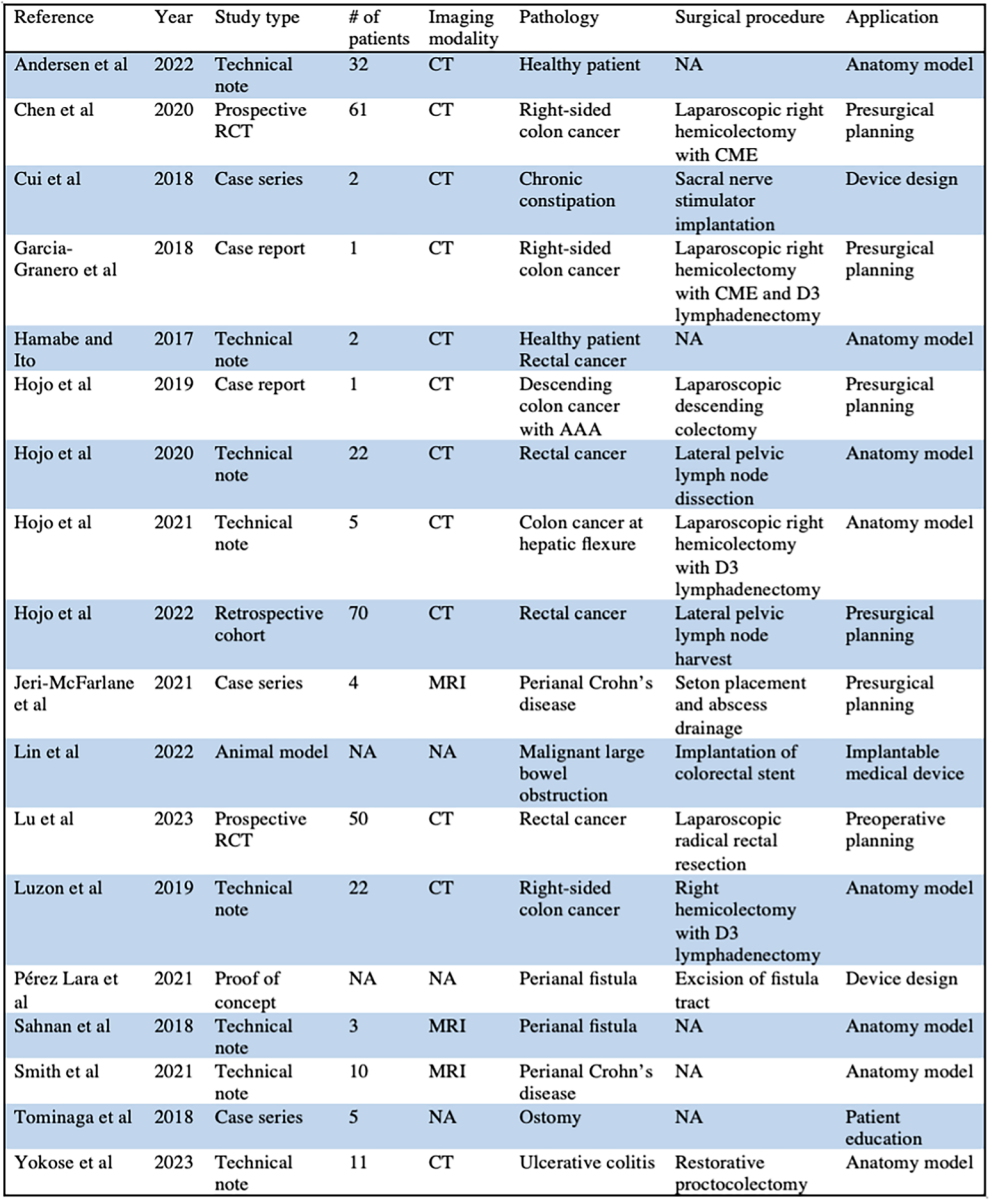
Summary of characteristics of the studies included in this review

For the purposes of this review, the papers were divided into the following categories: advanced surgical planning/device manufacturing (3), pre-surgical planning (6), pelvic anatomy model (2), vascular anatomy model (3), fistula anatomy model (2), ileal pouch anatomy model (1), and patient education (1). A summary of study characteristics and findings can be found in Table 2.

**Table 2:**
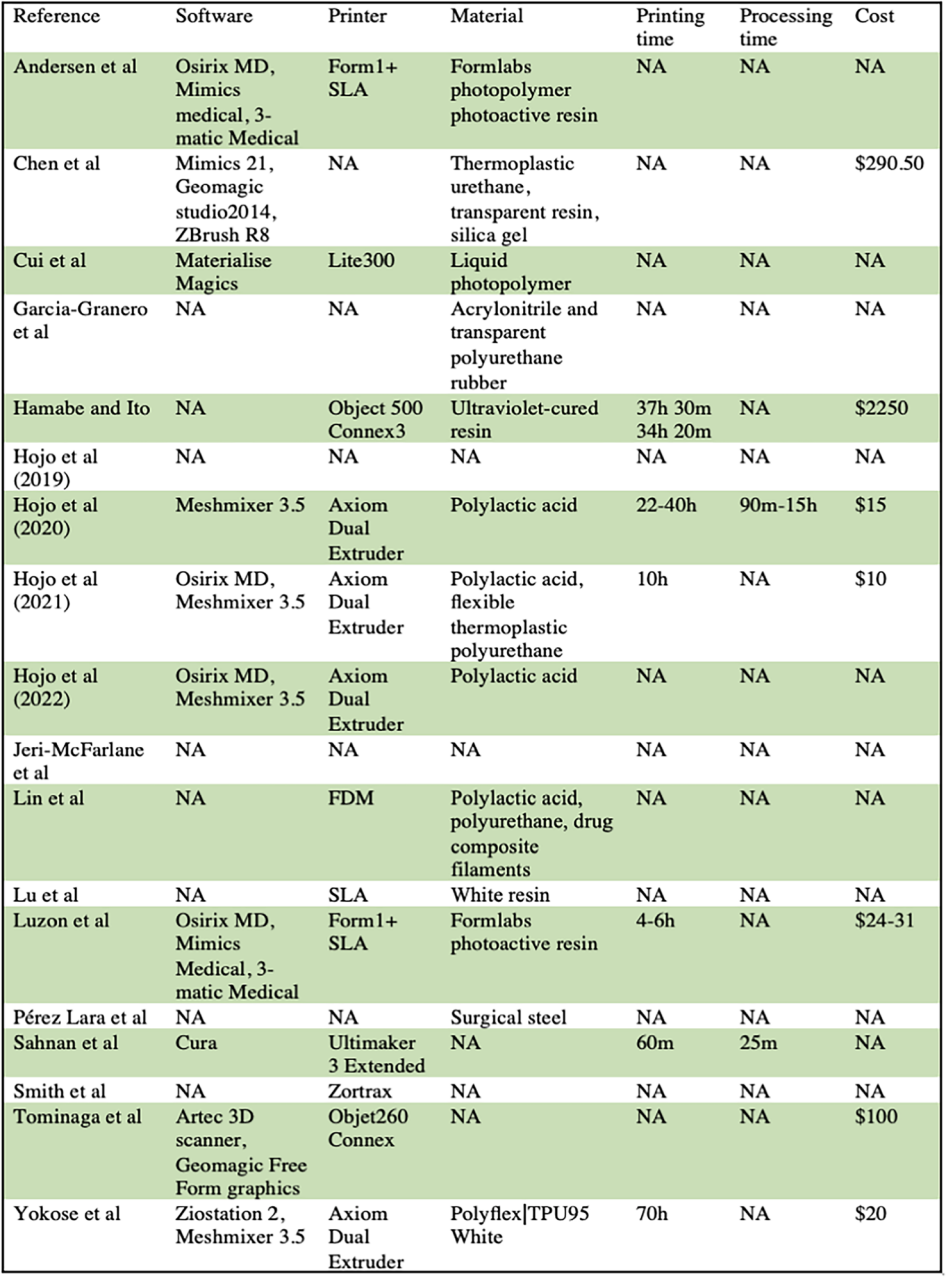
Information regarding printing process and materials. Printing time, production time, and cost are reported per individual model

*CT* computed tomography; *RCT* randomized controlled trial; *CME* complete mesocolic excision; *AAA* abdominal aortic aneurysm; *MRI* magnetic resonance imaging

### Advanced surgical planning and device manufacturing

Cui et al. created a 3D printed guiding device for sacral nerve stimulator implantation for two patients [10]. Segmentation was performed with Materialise Magics 3D Print Suite (Leuven, Belgium) using CT images and models were printed on a Lite300 3D printer (Uniontech, Shanghai, China) with liquid photopolymer (Somos XC11122, Heerlen, Netherland) as the printing material. The models were comprised of two parts: the basement part, which was flat template with a mild curve that matched the contour of the buttocks surface and included holes positioned over the target anatomy, and the cylinder part, which included hollow cylinders designed to encircle the test needles. The 3D printed models were used to perform sacral nerve stimulation in two patients. In both cases, the stimulator was placed at the target on the first attempt, operative time was < 20 min, no complications were reported, and patients reported > 50% symptom relief.

Lin et al. 3D printed colorectal stents for malignant large bowel obstruction in animal models [11]. These self-expandable stents were designed to mimic octopus suckers, tree frog toe pads, and gecko feet due to their adhesive properties. The stents were 3D printed on a fused filament fabrication printer (Allcct, Wuhan, China) using polylactic acid/polyurethane/drug composite filaments. Compared to stents without bioinspired microstructures, these bioinspired stents demonstrated better antimigration ability. The stents were tested and found to function as effective drug carriers. Additionally, their photothermal performance was evaluated and the stents were shown to induce hyperthermic tumor ablation in vitro and in vivo. Transanal placement of the stents was performed in rabbits successfully and easily. These authors describe the promising potential of 3D printed bioinspired colorectal stents to restore the size of the intestinal lumen, reduce the possibility of stent migration, and possibly deliver pharmacologic and hyperthermic therapies.

Pérez Lara et al. used 3D printing to create a specific curette for use in surgical treatment of perianal fistula [12]. As a proof of concept, they created a curette composed of two lateral rings through which suture could be threaded, allowing the curette to be pulled back and forth along the entire tract, and a central structure of discs in a stacked radial arrangement, allowing for 360° resection of fibrinous tissue. The instruments were printed using surgical steel. The authors report an increase in success rate of fistula sealing from 67 to 88% with use of their 3D printed instrument in conjunction with fibrin plugging as compared to fibrin plugging alone.

### Pre-surgical planning

Hojo et al. wrote a case report detailing the application of 3D printing in the care of an 89-year-old male with recurrent abdominal aortic aneurysm (AAA) after prior endovascular aneurysm repair (EVAR) and descending colon cancer which was diagnosed incidentally as part of workup for AAA revision [13]. CT scans were used to create 3D printed models of the AAA and colon with sculpted skin cover mimicking pneumoperitoneum in order to evaluate the safety of the procedure. The model revealed to the surgical team that using standard port placement they were unable to maneuver their instruments in a way that would allow them to perform the surgery. Therefore, they used the 3D printed model to attempt a lateral approach with specific port placements, which did allow for proper maneuvering. In the operating room, the ports were arranged to match the 3D model simulation and the descending colectomy was completed without complication. This paper illustrates the capability of presurgical planning using 3D printed models to alter surgical approach in a way that allows for safer procedures.

Garcia-Granero et al. created a 3D printed model for presurgical planning for a male patient with colon cancer scheduled for right hemicolectomy [14]. These authors’ particular focus was on replicating relevant vascular anatomy in hopes of decreasing the risk of bleeding while performing complete mesocolic excision (CME) with D3-lymphadenectomy. A laparoscopic colorectal surgeon delineated structures using CT scans to create 3D models of the organs and vascular structures encountered during gastrocolic trunk of Henle dissection and D3-lymphadenectomy. The model was printed using acrylonitrile to reproduce the arterial and portal systems and transparent polyurethane rubber to reproduce the stomach, duodenum, and pancreas. Surgery was planned based on the model and ultimately a laparoscopic right hemicolectomy with CME and high ligation of the ileocolic, right colic, and right branch of the middle colic vessels was performed, including D3-lymphadenectomy with dissection of the gastrocolic trunk of Henle and the surgical trunk of Gillot. Operative time was 190 min and no intraoperative bleeding or complications were reported. The patient was discharged on postoperative day four after an uncomplicated course and R0 resection was achieved with 24 lymph nodes detected. This study highlights the use of 3D printed models to plan and perform a safe and uncomplicated colonic resection.

Chen et al. conducted a prospective randomized controlled trial including 61 patients with right-sided colon cancer set to undergo laparoscopic right hemicolectomy with CME who were randomized to 3D printed, 3D image, or control groups [15]. In the 3D printed and 3D image groups, CT angiography was used to perform a 3D reconstruction using Materialise Mimics 21 software (Materialise, Belgium). Geomagic studio 2014 (3D System, America) and ZBrush R8 (Pixologic, America) were used for post processing. In the 3D printed group, the blood vessels were printed using thermoplastic urethane (TPU), the right hemicolon was printed using transparent resin, and the tumor was printed using silica gel. Materials cost per printed model was $290.50. The study found that duration of surgery and bleeding volume were significantly lower in the 3D printed and 3D image groups compared to the control group; these metrics were also significantly lower in the 3D printed group compared to the 3D image group. Number of lymph nodes dissected was significantly higher in the 3D printed and 3D image groups compared to the control group. These benefits continued to be seen to variable extents when surgeon experience was taken into account. Additionally, the medical expenses of the 3D printed group were significantly less than that of the control group and patients in the 3D printed group reported higher satisfaction with effective communication compared to those in the 3D image and control groups. This prospective randomized controlled trial regarding 3D printing in colorectal surgeries shows great promise for the field.

Lu et al. conducted a prospective randomized controlled trial to investigate the effects of presurgical planning with 3D printed models on perioperative outcomes in laparoscopic radical resection of rectal cancer [16]. The study enrolled 50 patients with rectal cancer scheduled to undergo laparoscopic radical resection, and the patients were randomly divided into a 3D model and control group. There were no significant differences in patient characteristics between the groups. In the 3D printed model group, CT images were used to create pelvic models that contained vasculature, bony structures, ureters, and the tumor with surrounding rectal tissue. These models were printed in resin white material by high-precision stereolithography (SLA) photocuring process and were used for preoperative planning. The study reports a statistically significant reduction in operative time, intraoperative blood loss, intraoperative time to locate the inferior mesenteric artery (IMA), intraoperative time to locate the left colic artery, and length of hospital stay. The authors conclude that 3D printed pelvic models have the potential to improve perioperative outcomes.

Hojo et al. performed a retrospective study using a propensity matched analysis to assess surgical outcomes after utilization of a 3D printed pelvic model for presurgical planning in lateral lymph node harvest [17]. One hundred and fifteen patients who had previously undergone lateral pelvic lymph node dissection for colorectal cancer were enrolled, some of whom had 3D printed models made preoperatively for surgical planning. After applying exclusion criteria and performing propensity matching, 35 patients each were assigned to the 3D printed and control groups. There were no significant differences between group characteristics. The 3D models were made from CT images using Osirix MD (Pixmeo Sarl, Bernex, Venice, CA) and Meshmixer 3.5 (Autodesk Inc, Venice, CA). The models were printed with polylactic acid on an Axiom Dual Extruder 3D printer (Airwolf 3D, Fountain Valley, CA) and included bone, vessels, muscles, nerves, and lymph nodes. The authors report significantly higher number of harvested lateral pelvic lymph nodes in the 3D model group than in the control group.

Jeri-McFarlane et al. report a case series of 3D printed models used for preoperative planning in four patients with perianal Crohn’s disease complicated by abscess [18]. MRI images were used to create 3D models with an artificial intelligence algorithm, and these models were used for planning prior to and reference during the surgical procedures, which included the placement of setons and drainage of abscesses. The authors recorded whether the internal fistula orifice(s) was or were located in the area indicated by the 3D reconstruction, if seton placement in the main fistula tracts was possible, and if abscesses were adequately drained. In all four cases, setons were successfully placed in the fistula tracts, the internal fistula orifice was localized, and the abscess was adequately drained, as confirmed by postoperative MRI. The authors concluded that 3D reconstruction of complex fistulas aids surgeons in identification of secondary fistula, internal anal orifices, and occult/deep abscess, which may otherwise be challenging to identify on MRI alone.

### Pelvic anatomy model

Hamabe and Ito created 3D printed models from CT scans of two patients: a healthy male volunteer and a female with rectal cancer [19]. A colorectal surgeon identified pelvic bone, muscles, internal and iliac vessels and their branches, nerves, and urogenital organs for segmentation. The models were printed on an Object500 Connex3 3D printer (Stratasys, Eden Prairie, MN, USA) using ultraviolet-cured resin as the printing material. The cost was $2250 per model. Print time per model was 37 h 30 min for the male model and 34 h 20 min for the female model. The models were able to be cleaved sagitally along the midline to facilitate visualization. These models were compared with laparoscopic surgical images and found by the authors to be reliable.

Hojo et al. manufactured 3D printed models preoperatively from CT scans of 22 patients who underwent lateral pelvic lymph node dissection for rectal cancer [20]. The images were segmented to create a virtual model that included relevant anatomical structures, and Meshmixer 3.5 (Autodesk Inc., Venice, CA, USA) was used to repair missing or damaged structures in the initial 3D image. The models were printed with white polylactic acid of 2.85 mm diameter using an Axiom Dual Extruder 3D Printer (Airwolf 3D, Fountain Valley, CA, USA). The cost was approximately $15 per model. Printing time decreased from 40 to 22 h between the first and last case. Thirty colorectal surgeons subjectively evaluated virtual and printed models and scored them in several areas based on a 5-point Likert scale. The mean score for utility of the models in understanding anatomy was 4.68 overall, 4.79 in cases with lateral pelvic node (LPN) metastasis, and 4.38 in cases without LPN metastasis. The study subjects showed a statistically significant preference for printed models over virtual models in terms of spatial comprehension and ease of use.

### Vascular anatomy model

Andersen et al. created 3D printed models from 32 CT scans to assess the spatial relationship of variations in mesenteric vascular anatomy [21]. Osirix MD (Pixmeo, Bernex, Switzerland), Mimics Medical (Materialise NV, Leuven, Belgium), and 3-matic Medical (Materialise NV, Leuven, Belgium) software were used for segmentation image processing. Parameters including distances between arterial origins; the ileocolic artery (ICA), middle colic artery (MCA), and right colic artery (RCA); the distance from the MCA origin to its bifurcation; and vessel caliber were measured on the virtual models. The models were printed in cases of unusual or complex anatomy that was not well understood with virtual models alone. Such models were printed using Form1 + SLA printer (Formlabs, Sommerville, MA, USA) onto Formlabs photopolymer photoactive resin. Using virtual and physical models, the authors determined that MCA bifurcation was left of the superior mesenteric vein (SMV) in 4 (12.1%), in front of SMV in 17 (53.1%) and right of SMV in 11 (34.4%) models. Median distance from the MCA origin to bifurcation was 3.21 (1.18–15.60) cm and accessory MCA occurred in 31.3% of models. The authors conclude that virtual and printed 3D models allowed for detailed assessment of the multiple variations of mesenteric vascular anatomy.

Luzon et al. also assessed mesenteric vascular anatomy using 3D printing [22]. In this paper, 22 patients undergoing right hemicolectomy with D3 lymphadenectomy had CT scans rendered to 3D models using Osirix MD (Pixmeo, Bernex, Switzerland), Mimics Medical (Materialise NV, Leuven, Belgium), and 3-matic Medical (Materialise NV, Leuven, Belgium) software. The Form1 + SLA printer (Formlabs, Sommerville, MA, USA) was used to print the models onto Formlabs photoactive resin. Total material cost per model was $24–31 and 4–6 h was needed to print each model. Four parameters measured: distance between the origins of the ileocolic and the middle colic artery, distance between the termination of the gastrocolic trunk and the ileocolic vein, and the calibers of the middle colic and ileocolic arteries. When compared to perioperative measurements, there was strong correlation between interarterial distances and weak correlation between intervenous distances. This paper demonstrates the value as well as current limitations of using 3D printing for mesenteric vascular modeling.

Hojo et al. 3D retrospectively printed deformable vascular models for patients who underwent laparoscopic right hemicolectomy [23]. Five patients who had previously undergone laparoscopic right hemicolectomy with D3 lymphadenectomy for colon cancer at the hepatic flexure were enrolled in the study. Using CT images, segmentation was performed using Osirix MD software (Pixmeo Sarl, Bernex, Switzerland) and modifications were performed using Meshmixer 3.5 software (Autodesk Inc., Venice, CA, USA). The images were printed on an Axiom Dual Extruder 3D printer (Airwolf 3D, Fountain Valley, CA, USA), using polylactic acid for the pancreas and duodenum and flexible thermoplastic polyurethane for the superior mesenteric artery (SMA) and SMV and their branches. Average printing time was ten hours with time decreasing with each subsequent model, and the average materials cost per model was $10. The authors retrospectively compared the individual deformable 3D models with the intraoperative views of the arrangement of the vessels during the high tying of the main vasculature, obtained from intraoperative videos, and found the models to be accurate. The deformable model proved useful in replicating the changes in spatial arrangement of the superior mesenteric vasculature caused by transverse colon mobilization.

### Fistula anatomy model

Sahnan et al. used MRI to construct 3D printed models of complex perianal fistulas [24]. Segmentation was performed from T2-weighted MRI sequences by a gastrointestinal radiologist. The files were prepared using Cura software (Ultimaker Cura 3.0.4, Ultimaker B.V., 4191 PN Geldermalsen, The Netherlands) and printed on an Ultimaker 3 Extended 3D printer. Animation was also created in collaboration with Touch Surgery™. Each model took approximately 60 min to make. Three models were created: a trans-sphincteric fistula with infralevator extension, a trans-sphincteric fistula with a horseshoe, and a complex trans-sphincteric and intersphincteric fistulas. This paper demonstrates the feasibility of 3D printing perianal fistula models.

Smith et al. used MRI to create 3D printed models of complex perianal Crohn’s disease [25]. Ten patients who had previously undergone pelvic MRI for perianal Crohn’s disease were selected for 3D modeling due to complexity of their fistulas. Seed-based region growing was employed to segment 3D images from T2-weighted MRI sequences, and these images were printed on a Zortrax printer (Zortrax, M200, Poland). Five experienced colorectal surgeons were given summaries of the clinical scenarios and 2D images and from this information drew their perceived anatomy and proposed an operative plan. These surgeons were then presented with 3D images and models of the same patients and recorded changes to their anatomical assessment and operative plan. The 3D images and models resulted in a change in anatomical interpretation in 50% of assessments (a change was defined as 4 out of 5 surgeons changing their anatomic interpretation after interacting with the 3D models). The authors reported that all participants agreed that the 3D images/models made interpretation of the anatomy much faster and would be useful in their clinical practice.

### Ileal pouch anatomy model

Yokose et al. used 3D printing to create a preoperative simulation of ileal pouch-anal anastomosis in patients with ulcerative colitis (UC) who had previously undergone restorative proctocolectomy [26]. CT scans of six patients who had received hand sewn ileal pouch-anal anastomosis (IAA) and 5 patients who had received stapled ileal pouch-anal canal anastomosis (IACA) were used to create 3D printed models. The models were reconstructed using Ziostation 2 (Ziosoft) and Mexmisher 3.5 (Autodesk Inc) software and were printed on an Axiom Dual Extruder 3D printer (Airwolf 3D) using deformable PolyFlex TPU95 White 2.85 mm (Polymaker) material. The materials cost about $20 per model and the printing time was about 70 h per model. Using these models, several arterial distances were measured, and it was determined that the distance between SMA root and tip of ileal artery was longer in IAA group than IACA group. Additionally, distance from tip of ileal artery to coccyx and to lower edge of pubis were longer in IACA group than IAA group. This study demonstrates the correlation between anatomic vasculature distances, as measured by 3D printed models, with the type of ileal pouch anastomosis created in patients who had previously undergone restorative proctocolectomy for UC.

### Patient education

Tominaga et al. created 3D stoma models and face plates for patient education about stoma care [27]. 3D graphics for each stoma were created using an Artec 3D scanner (Artec, Luxembourg, Luxembourg), customized with Geomagic Free Form (Geomagic, Cary, N.C., USA) graphics, and printed with an Objet260 Connex printer (Stratasys, Eden Prairie, Minn., USA). Five patients used the models to practice stoma care, troubleshooting problems with the stoma, and cutting their own plates. Cost was about $100 per patient and the models took several days to create. All patients reported understand problems related to their stomas and became self-reliant in stoma care. This case series exemplifies the utility of 3D printing technology in educating patient about their own anatomy and disease processes.

## Discussion

This systematic review provides an overview of the current clinical applications of 3D printing in the field of colorectal surgery. A thorough literature search revealed a small number of studies (18) that met inclusion criteria, and of these studies, the majority were technical notes, feasibility studies, or case reports. This indicates that 3D printing in colorectal surgery is in its early stages, and the papers included in this review suggest that there is significant potential for increased application of the technology within the field.

Lack of familiarity with or access to the technology, cost, and production time have all contribute to the slow implementation of 3D printing in colorectal surgery [5]. Cost per model reported in these papers ranged from $10 to 100 with one outlier of $2250, in which ultraviolet cured resin was used as the printing material [19, 20, 22, 23, 26]. There is of course the additional cost of the printer itself and the time and salary of the staff involved in production. To alleviate financial stain, recent efforts have included current Medicare experimental codes for potential future payments as it relates to the reimbursement for the use of 3D printing and other advance manufacturing approaches at the point of care [28, 29]. Production time varied widely, largely due to the discrepancy in whether pre- and postproduction processing were included with print time, but estimates ranged from one hours to several days per model [19, 20, 22–24, 26, 27]. Several studies reported that with each iteration, production time became progressively shorter [20, 23]. Others note that the actual print time is an automated process which does not require active contribution from or the constant presence of the production team [26]. Both cost and production time are of course in part determined by the complexity of the model and the materials used. Traditional 3D models were rigid, with poor ability to mimic living tissue [30]. However, advancements continue to be made in 3D printing material, and the recent creation of deformable models suggests that 3D printed models will only become more realistic with time [23, 26].

The papers included in this review describe various applications for 3D printing in abdominopelvic colorectal surgery, although only five such studies included in this review actually use their models in a clinical setting [13–17]. These papers include two prospective randomized controlled trials, two case reports, and one retrospective outcomes study. The two case reports demonstrated favorable outcomes, with one even describing a change in surgical approach preoperatively after working with the 3D model [13, 14]. The retrospective outcomes study is propensity matched and identifies improvement in lymph node harvesting when 3D models are examined preoperatively [20]. Chen’s randomized controlled trial is a well-designed study which showed statistically significant benefit for operative time, blood loss, number of lymph nodes dissected, and medical cost in 3D model groups compared to the control group [15]. Lu’s similarly impactful study documented equally impressive outcomes, including significantly reduced operative time, blood loss, and hospital length of stay [16]. While these are the only two studies of their kind currently in the literature, they suggests great promise for the application of 3D printing in colorectal clinical practice. Additional prospective randomized controlled trials in the future would help solidify this conclusion. The rest of the studies describing clinical applications of 3D printing in abdominal colorectal surgery simply discuss the feasibility and accuracy of 3D printing in creating anatomical models [19–26]. Specific anatomical focuses included pelvic anatomy, vascular anatomy, perianal fistula, and ileal pouch. While several of these studies describe surgeon satisfaction with these models in a theoretical setting, they do not prove the clinical application of 3D printing in colorectal surgery.

There are few studies that evaluate the role of 3D printing in anorectal surgery, and those included in this paper focus specifically on perianal fistula. In only two of these four papers were 3D printed models used for clinical applications; the other two reported ability to create models and surgeons’ evaluation of the models’ potentially utility [18, 24, 25]. One of these papers was a proof of concept which reported favorable outcomes but lacked granularity of detail in terms of patient outcomes [12]. While perianal fistulas are often diagnosed by physical exam alone, imaging modalities currently used to evaluate fistulas with complex anatomy include MRI and endoanal ultrasound. A 2012 meta-analysis reports sensitivities of 0.87 for both of these modalities and specificities of 0.69 and 0.43 for MRI and endoanal ultrasound, respectively [31]. The paper notes that both specificity values are considered to be diagnostically poor. In light of this, there seems to be a role for 3D printing in better understanding complex perianal fistula anatomy although further studies are warranted to confirm its efficacy. To date, 3D printing for other anorectal pathologies, such as hemorrhoids, anal tumors, and anal fissures, has not been studied.

Even more sparse are publications related to 3D printing and advanced surgical planning or device design. Of the three papers discussed in this review, two use 3D printed devices to treat human subjects, with the devices in these cases being a 3D printed, patient-specific guidance system to facilitate sacral nerve stimulation and a 3D printed curette for excision of perianal fistula tracts [10, 12]. The other device described in this review is a 3D printed colorectal stent intended for use in obstructing colon masses, however the model has only been tested in animals thus far [11]. While these technologies show promise the potential utilization of 3D printing in advanced surgical planning and device design in the field of colorectal surgery has been largely untapped.

There was one study included in this review that examined the value of 3D printing specifically for patient education; this article detailed the benefit of 3D printed stoma models on patient’s ability to become self-reliant with stoma care [27]. Chen et al. also commented on how being shown 3D models of their anatomy increased patient’s satisfaction with effective communication, although this was a secondary outcome [15]. Beyond this, there is a paucity of literature related specifically to 3D printing for patient education in colorectal surgery. In other surgical subspecialties such as orthopedics, pediatric surgery, and urology, 3D printing has been used for the explicit purpose of patient education with statistically significant improvements in patient understanding of their disease processes and surgical procedures [32–35]. Surely similar strides could be made in the field of colorectal surgery.

While this review did not include papers regarding 3D printing in student/trainee education, there have been several such studies in the field of colorectal surgery that have shown promise. 3D printed models have been shown to improve interns’ understanding of gastrocolic trunk anatomy, to serve as an informative adjunct to medical students’ cadaver lab, and to improve surgical residents’ understanding of perianal fistula anatomy [36–38]. In addition, several 3D printed small and large bowel anastomosis simulators have been studied for use by junior residents, senior residents, and attendings [39–41]. Participants in these studies largely considered the models useful for educational purposes, although they noted areas for improvement in product quality related to similarity to actual bowel.

There were several limitations of this review paper which should be addressed. As previously noted, there is a paucity of literature describing clinical applications 3D printing in colorectal surgery. From this already small pool, most publications are feasibility studies or technical notes, which do not directly examine clinical application. Of the papers included in this review, only four actually employed their models for use in the operating room. Only one study in this review was a prospective randomized controlled trial. Additionally, as many papers omit cost in their analysis, it is difficult to determine the value proposition of these clinical applications. The low level of evidence provided by the majority of papers included in this review does very little to directly prove the utility of 3D printing in colorectal surgery.

There are several potential future directions for this technology. Of foremost importance is the addition of prospective randomized controlled trials to the literature in order to quantitatively demonstrate the benefits of 3D printing in colorectal surgery. There is much opportunity to conduct these studies within the domain of presurgical planning. Such trials should compare objective variables such as cost, printing/processing time, operative time, estimated blood loss, and intraoperative complications between 3D printed and control groups. Regarding case reports and case series, much of the currently literature omits such quantifiable data as mentioned above, which are crucial to understanding the advantages of the technology. If future case reports and case series were to include more objective evidence, this would strengthen the body of literature on 3D printing in colorectal surgery. Additionally, there may be value in focusing the technology on areas of particular anatomic intricacy, such as complex anorectal pathology or aberrant vasculature, in order to achieve maximum benefit. Finally, there seems to be great potential for the use of 3D printing for patient education in colorectal surgery, and the scarceness of literature on this topic leaves room for future studies to expand on this technology’s ability to improve patient communication and understanding.

## Conclusion

This systematic review assesses the current state of literature regarding the clinical applications of 3D printing in colorectal surgery. This review is comprised mostly of feasibility studies and technical notes, which highlight the ability 3D models to demonstrate complex spatial relationships. There also appears to be a benefit regarding patient understanding and education. Whether 3D printing ultimately impacts outcomes in colorectal surgery is yet to be seen, as this was only adequately studied by one paper in this review, in which results were favorable. While the literature to date is in need of higher level studies to prove the clinical utility of 3D printing in colorectal surgery, the findings of this review do suggest many promising applications within the field with the potential to improve patient outcomes and experiences.

## Data Availability

No datasets were generated or analysed during the current study.

## Appendix A

### PRIMSA checklist

**Table:**

		Reporting Item	Page Number
Title			
Title	#1	Identify the report as a systematic review	4
Abstract			
Abstract	#2	Report an abstract addressing each item in the PRISMA 2020 for Abstracts checklist	2
Introduction			
Background/rationale	#3	Describe the rationale for the review in the context of existing knowledge	4–5
Objectives	#4	Provide an explicit statement of the objective(s) or question(s) the review addresses	5
Methods			
Eligibility criteria	#5	Specify the inclusion and exclusion criteria for the review and how studies were grouped for the syntheses	6
Information sources	#6	Specify all databases, registers, websites, organisations, reference lists, and other sources searched or consulted to identify studies. Specify the date when each source was last searched or consulted	5–6
Search strategy	#7	Present the full search strategies for all databases, registers, and websites, including any filters and limits used	5–6
Selection process	#8	Specify the methods used to decide whether a study met the inclusion criteria of the review, including how many reviewers screened each record and each report retrieved, whether they worked independently, and, if applicable, details of automation tools used in the process	6
Data collection process	#9	Specify the methods used to collect data from reports, including how many reviewers collected data from each report, whether they worked independently, any processes for obtaining or confirming data from study investigators, and, if applicable, details of automation tools used in the process	6–7
Data items	#10a	List and define all outcomes for which data were sought. Specify whether all results that were compatible with each outcome domain in each study were sought (for example, for all measures, time points, analyses), and, if not, the methods used to decide which results to collect	6–7
Study risk of bias assessment	#11	Specify the methods used to assess risk of bias in the included studies, including details of the tool(s) used, how many reviewers assessed each study and whether they worked independently, and, if applicable, details of automation tools used in the process	6
Effect measures	#12	Specify for each outcome the effect measure(s) (such as risk ratio, mean difference) used in the synthesis or presentation of results	n/a
Synthesis methods	#13a	Describe the processes used to decide which studies were eligible for each synthesis (such as tabulating the study intervention characteristics and comparing against the planned groups for each synthesis (item #5))	6–7
Synthesis methods	#13b	Describe any methods required to prepare the data for presentation or synthesis, such as handling of missing summary statistics or data conversions	6–7
Synthesis methods	#13c	Describe any methods used to tabulate or visually display results of individual studies and syntheses	Tables 1 and 2
Synthesis methods	#13d	Describe any methods used to synthesise results and provide a rationale for the choice(s). If meta-analysis was performed, describe the model(s), method(s) to identify the presence and extent of statistical heterogeneity, and software package(s) used	n/a- results were not synthesized into individual recommendations due to paucity of evidence
Synthesis methods	#13e	Describe any methods used to explore possible causes of heterogeneity among study results (such as subgroup analysis, meta-regression)	n/a- results were not synthesized into individual recommendations due to paucity of evidence
Synthesis methods	#13f	Describe any sensitivity analyses conducted to assess robustness of the synthesised results	n/a- results were not synthesized into individual recommendations due to paucity of evidence
Reporting bias assessment	#14	Describe any methods used to assess risk of bias due to missing results in a synthesis (arising from reporting biases)	n/a- results were not synthesized into individual recommendations due to paucity of evidence
Certainty assessment	#15	Describe any methods used to assess certainty (or confidence) in the body of evidence for an outcome	n/a- results were not synthesized into individual recommendations due to paucity of evidence
Data items	#10b	List and define all other variables for which data were sought (such as participant and intervention characteristics, funding sources). Describe any assumptions made about any missing or unclear information	6–7
Results			
Study selection	#16a	Describe the results of the search and selection process, from the number of records identified in the search to the number of studies included in the review, ideally using a flow diagram (http://www.prisma-statement.org/PRISMAStatement/FlowDiagram)	7–10, Fig. 1
Study selection	#16b	Cite studies that might appear to meet the inclusion criteria, but which were excluded, and explain why they were excluded	25–26, Fig. 1
Study characteristics	#17	Cite each included study and present its characteristics	10–20
Risk of bias in studies	#18	Present assessments of risk of bias for each included study	10–20
Results of individual studies	#19	For all outcomes, present for each study (a) summary statistics for each group (where appropriate) and (b) an effect estimate and its precision (such as confidence/credible interval), ideally using structured tables or plots	10–20
Results of syntheses	#20a	For each synthesis, briefly summarise the characteristics and risk of bias among contributing studies	n/a- results were not synthesized into individual recommendations due to paucity of evidence
Results of syntheses	#20b	Present results of all statistical syntheses conducted. If meta-analysis was done, present for each the summary estimate and its precision (such as confidence/credible interval) and measures of statistical heterogeneity. If comparing groups, describe the direction of the effect	n/a- results were not synthesized into individual recommendations due to paucity of evidence
Results of syntheses	#20c	Present results of all investigations of possible causes of heterogeneity among study results	n/a- results were not synthesized into individual recommendations due to paucity of evidence
Results of syntheses	#20d	Present results of all sensitivity analyses conducted to assess the robustness of the synthesised results	n/a- results were not synthesized into individual recommendations due to paucity of evidence
Risk of reporting biases in syntheses	#21	Present assessments of risk of bias due to missing results (arising from reporting biases) for each synthesis assessed	n/a- results were not synthesized into individual recommendations due to paucity of evidence
Certainty of evidence	#22	Present assessments of certainty (or confidence) in the body of evidence for each outcome assessed	n/a- results were not synthesized into individual recommendations due to paucity of evidence
Discussion			
Results in context	#23a	Provide a general interpretation of the results in the context of other evidence	22–27
Limitations of included studies	#23b	Discuss any limitations of the evidence included in the review	26
Limitations of the review methods	#23c	Discuss any limitations of the review processes used	26
Implications	#23d	Discuss implications of the results for practice, policy, and future research	26–27
Other information			
Registration and protocol	#24a	Provide registration information for the review, including register name and registration number, or state that the review was not registered	n/a- review not registered
Registration and protocol	#24b	Indicate where the review protocol can be accessed, or state that a protocol was not prepared	n/a- protocol was not prepared
Registration and protocol	#24c	Describe and explain any amendments to information provided at registration or in the protocol	n/a
Support	#25	Describe sources of financial or non-financial support for the review, and the role of the funders or sponsors in the review	3
Competing interests	#26	Declare any competing interests of review authors	3
Availability of data, code, and other materials	#27	Report which of the following are publicly available and where they can be found: template data collection forms; data extracted from included studies; data used for all analyses; analytic code; any other materials used in the review	n/a- not publicly available

## Declarations

The authors did not receive support from any organization for the submitted work.
